# The implications of statin induced peripheral neuropathy

**DOI:** 10.1186/1757-1146-4-S1-P57

**Published:** 2011-05-20

**Authors:** Brenton West

**Affiliations:** 1 Peninsula Health, Melbourne, Victoria, 3930, Australia

## 

Peripheral neuropathy is a neurological condition that commonly presents to the practicing podiatrist. Traditionally when sensory neuropathy is present one assumes diabetes mellitus to be the contributing aetiology. However, this is not always the case. Other common causes such as spinal damage, alcoholism, and vitamin B12 deficiency can be precipitating aetiologies that the clinician would be aware. 26% of individuals over 65 years of age (without a predisposing disease known to cause neuropathy) will develop peripheral neuropathy at some point in life. What does the clinician do when none of the common causes are present? One clinical presentation tested this predicament. A female patient presented with diagnosed statin induced sensory peripheral neuropathy. This uncommon aetiology lead to a literature search relating to the less common causes of peripheral neuropathy. There are reported cases of statin induced peripheral neuropathy, which in the initial stages can be reversible. Accumulating this research is difficult due to the wide variances in results and study methods; proving further independent research is needed. The reported incidence rate of peripheral neuropathy ranges between 4-14 times more likely for those on statins compared to control groups. This case study will help to raise the profile of this aetiology with the aim to preventing misdiagnosis. Interestingly, the quality of life and political implications of statin induced peripheral neuropathy are an aspect not commonly considered. What was the eventual outcome and how was the patient's quality of life affected? The patient now suffers from an irreversible peripheral neuropathy. Thus, the patient is now exposed to the complications of peripheral neuropathy, including shoe wear and hosiery changes, daily foot checks, pressure area development, and possibility of future neuropathic ulceration. Could the outcome of peripheral neuropathy have been changed if podiatry was involved earlier in the patient's management? The early detection of peripheral neuropathy may have lead to a change in specific medication to a statin that is not associated with peripheral neuropathy, therefore preventing the irreversible damage. It should be asked was this patient managed appropriately? Without the statins the patient would be at risk of hypercholesterolemia, therefore increasing cardiovascular risks such as acute myocardial infarction. Diet and exercise modifications can have great effect with lowering serum cholesterol. Such modifications should be the initial treatment, or should be utilised in conjunction with pharmacological intervention. Statins should be used to lower serum cholesterol levels when unable to do so with dietary modifications. However, there are alternative statins available which do not contain the same side effects (peripheral neuropathy) as others within the class. So the drug of choice should be made critically. Often medication is the first line treatment option due to patient compliance and ease of medical practitioner review. But it needs to be asked, who would be liable if the patient was to ulcerate and progressed to amputation? The patient, medical practitioner, or podiatrist?

